# I PLAY AT WORK—ten principles for transforming work processes through gamification

**DOI:** 10.3389/fpsyg.2014.00014

**Published:** 2014-01-30

**Authors:** Florin Oprescu, Christian Jones, Mary Katsikitis

**Affiliations:** ^1^Faculty of Science, Health, Education and Engineering, School of Health and Sport Sciences, University of the Sunshine CoastSippy Downs, QLD, Australia; ^2^Faculty of Arts and Business, University of the Sunshine CoastSippy Downs, QLD, Australia

**Keywords:** gamification, gamified workplaces, gamified systems, redesign of work processes, organizational and personal wellbeing, play, wellbeing

## Abstract

Gamified workplaces could be a positive and innovative solution to addressing contemporary problems in organizations. Such problems include high levels of stress, reduced sense of community, reduced loyalty and rapid changes in the workforce. To better prepare organizations for the future it may be helpful to identify and understand the potential advantages, disadvantages and areas for future research in relationship to the use of gamification for personal and organizational wellbeing. An analysis of research literature across disciplines in combination with expert opinion identified gamified workplaces as a promising strategy for promoting wellbeing. Furthermore, this paper proposes a set of 10 principles (I PLAY AT WORK) that may support gamification efforts. In addition to the value of mapping the present for the benefit of the future, there is also considerable value in reshaping core ideas related to the workplaces. Gamified workplaces can provide opportunities for a more vigorous and strategic inter-disciplinary research agenda that can stimulate investments in the area.

## Introduction

Recently it was proposed that gamified services will become a key element in marketing and customer retention initiatives (Dorling and McCaffery, 2012). We are proposing that gamified workplaces will become a key element in the recruitment and retention of a productive and healthy workforce. This is because using gamified systems in the workplace could be a positive and innovative solution to addressing contemporary issues in organizations. Such issues include high levels of stress (Perryer et al., 2012), reduced social capital (Zhu et al., 2013), reduced loyalty and rapid changes in the workforce demographics (Dorling and McCaffery, 2012). From a health perspective, organizations will be likely to face an increased prevalence of stress, overweight and obesity in the workforce with a direct and indirect impact on ailments such as cardiovascular disease, diabetes and even addictions (Bosworth, 2012). To better prepare organizations for the future it may be helpful to consider the question: “What gaming principles could be used to gamify work places and processes so that both the employee and the employer benefit in terms of productivity and wellbeing?” In order to attempt to answer this question we will first discuss gamified workplaces, gamification, and gamification research.

Gamified workplaces are not workplaces where employees play videogames (Lewis, 2007; Farrington, 2011; Landers and Callan, 2011; Connolly et al., 2012; Popescu et al., 2012, 2013). Gamified workplaces can be defined as organizations that use gamification to transform some of their work processes into a game-like experience for the employees by applying selected principles of game design and game interaction. The long term goal of a gamified workplace is to increase wellbeing at the organizational level (i.e., productivity) and personal level (i.e., work satisfaction).

Gamification is a concept that garners increasing attention across funding bodies, academic disciplines and various industries (Dorling and McCaffery, 2012). For the purpose of this paper workplace gamification is defined as the adaptation and application of game design principles and game interaction elements to workplace processes and behaviors. Game interaction elements include both game mechanics and game dynamics. Game mechanics refer to the reward systems and game dynamics refer to the user progression that may lead to the rewards (Dorling and McCaffery, 2012).

Gamification research is still in its infancy and the transition from games to gamification remains a work in progress as documented by recent publications (Deterding et al., 2011; Koutropoulos, 2012; Dominguez et al., 2013; Rednic et al., 2013). In the literature, “gamification” has been described as the use of video-game elements to improve user engagement and experience with non-game initiatives (Deterding et al., 2011; Dominguez et al., 2013; Rednic et al., 2013). Hypothesized benefits include more engaging workplaces and additional opportunities for productive collaboration (Deterding et al., 2011; Rednic et al., 2013), increased motivation (Koutropoulos, 2012; Dominguez et al., 2013) and work place customization for enhanced personal control (Rednic et al., 2013).

There are many types of games applicable to workplaces. Games can be used for recruitment and training purposes (i.e., military), for lead generation, recruitment and public relations (i.e., Intelligence agencies), for selection (i.e., problem based interviewing), for training, continuous professional development and up skilling of the workforce (i.e., health professions), for planning, performance and review processes (i.e., public sector), for skill based promotion (i.e., engineering), and for development of personal health skills (i.e., Keas) among others. Crookall (2010) provided a list of disciplines using games and publication outlets for games and identified some of the research development needs in the field.

## I play at work—10 principles for workplace gamification

In response to the needs identified in previous research this section proposes 10 guiding principles that may facilitate the adoption and use of gamification in everyday workplace processes and not just in training or special engagement events. Such principles could be used to make workplace processes more appealing for employees and more value generating for employers. This is because, from a psychological perspective, gamified workplaces could be seen as self-improving, self-learning entities where behavior change is created and sustained (Baranowski et al., 2011).

The ten principles proposed for gamifying work processes have been organized under the mnemonic I PLAY AT WORK.

**Table:** 

**ID**	**Principle**	**Description**	**Theoretical basis**	**Expected benefits**
1.	I Orientation	Gamified processes place the user (employee) at the center of the experience	Operant conditioning, locus of control, self-efficacy	Increased engagement, sense of control and self-efficacy
2.	Persuasive elements	Gamified processes include persuasive elements based on sound psychological and behavioral theories	Theory of planned behavior, stages of change theory, uncertainty management	Adoption of new initiatives Increased satisfaction with internal communication
3.	Learning orientation	Focus on knowledge acquisition, skill development, motivational outcomes or behavior change	Theory of planned behavior, self-efficacy, experiential learning	Development of personal and organizational capabilities and resources
4.	Achievement based rewards	Focus on a justifiable and predictable return on investment	Theory of planned behavior, experiential learning	Increased personal satisfaction and employee retention
5.	Y Generation adaptable	Generation Y is the fastest growing segment of the workforce and they are looking for work experiences that are supportive, fun and engaging	Hierarchy of needs, psychogenic needs	Employee acquisition and retention
6.	Amusement factors	Inclusion of humor, play and fun elements as part of the work processes	Psychogenic needs, social learning theory	Increased personal satisfaction and enhanced wellbeing
7.	Transformative	Use of a balanced and attractive combination of competition and collaboration in order to transform existing work processes within an organization	Leadership theories, team building	Enhanced productivity
8.	Wellbeing oriented	Focus on personal and organizational wellbeing	Organizational behavior, self-competence	Enhanced personal and organizational wellbeing
9.	Research generating	Collaborative research efforts must be encouraged to justify future investments in the area	Organizational needs assessment and evaluation	Enhanced monitoring and decision making
10.	Knowledge-based	Based on knowledge, either as an outcome or as feedback	Organizational training, adult learning	Development of personal and organizational capabilities and resources

The aforementioned will be described next in conjunction with the supporting literature.

### I orientation

Successful games engage the users by focusing on cognitive, emotional, and social outcomes (Dominguez et al., 2013). Successful games include personally relevant, carefully designed and increasingly difficult challenges, often interspersed with humorous elements (Coller and Scott, 2009). Such challenges require increased cognitive effort, skill development and contributions from the users.

In the same way that games encourage users to progress through various levels, gamified work processes may encourage users to progress through increasingly difficult tasks (Deterding et al., 2011; Barthel, 2013; Popescu et al., 2013), may increase participation in special initiatives (Papastergiou, 2009; Barthel, 2013) may increase adoption of new programs (Lu et al., 2012; Rednic et al., 2013), and may increase program use period (Coller and Scott, 2009; Kato, 2010; Lu et al., 2012).

An interdisciplinary approach to system design is recommended for best results (Brox et al., 2011; Ahola et al., 2013). It is recommended to use a user-centered philosophy as a basis for process and system design (Brox et al., 2011; Bosworth, 2012; Cafazzo et al., 2012). For example, each user, in consultation with colleagues and managers, could set concrete goals and concrete standards of success or failure (Brox et al., 2011; Barthel, 2013). Such an approach may allow for various work processes to be tailored to a specific audience (Baranowski et al., 2012; Ahola et al., 2013; Barthel, 2013) while allowing for personalization based on individual preferences (Barreneche, 2012).

### Persuasive elements

Inclusion of persuasive elements based on sound psychological and behavioral theories may encourage participants to want to explore and learn more in ways that are beneficial to both the user and the company (Michaelides, 2011; Barreneche, 2012; Barthel, 2013). For example, the use of persuasive elements in communication and delivery of new initiatives could enhance lead generation and participant acquisition (Barreneche, 2012; Lease and Yilmaz, 2013).

An example is Google. At a very high level Google is using gamification principles to influence adult behavior (Barreneche, 2012). Through game like elements Google incentivizes users to contribute data (i.e., reviews and corrections), to change behavior (i.e., selection of a restaurant) or to have them pay for services (Google Ads). It also uses continuous passive and active push and pull of data to/from users. In this way, Google uses the benefits of crowdsourcing inherent in gamification to collect data in automated and non-automated ways (Lease and Yilmaz, 2013) with a focus on continuous learning and resource development.

### Learning orientation

An extensive review of games and their potential positive impact on learning is available elsewhere (Connolly et al., 2012). Some key benefits noted were knowledge acquisition, skill (motor, cognitive, social, and emotional) development, motivational outcomes and behavior change (Connolly et al., 2012). Gamification can support learning through immediate or delayed feedback (Crookall, 2010; Perryer et al., 2012; Popescu et al., 2012), resulting in increased self-efficacy (Papastergiou, 2009; Lu et al., 2012; Perryer et al., 2012; Popescu et al., 2013) and healthy behavior change (Kato, 2010; Lenihan, 2012; Kharrazi et al., 2012) Gamified work process design may be most successful if it is informed by psychological and health behavior theories (Brox et al., 2011; Baranowski et al., 2012), including those focused on rewards.

### Achievement based rewards

The use of virtual or real rewards has been shown to increase adoption of new initiatives (Perryer et al., 2012; Whitton, 2012; Dominguez et al., 2013). Achievement based rewards could also help build relationships between employees and stronger loyalty to the company if gamified systems and programs are perceived by employees as wellbeing oriented perks (Perryer et al., 2012; Dominguez et al., 2013), For example, a gamified process focuses on positive feedback as a form of virtual reward and as a way to increase self-efficacy (Papastergiou, 2009; Kark, 2011; Lu et al., 2012; Perryer et al., 2012). This is because the employees may expect a justifiable and predictable return on their investment (Bosworth, 2012).

### Y-generation adaptable

New employees, especially the Y generation, may find a gamified workplace more attractive because they value work experiences that are supportive, fun, engaging and rewarding (Bosworth, 2012). Y generation individuals want to express their opinions, want to try out different personalities in a safe environment and want to be rewarded often (even if only virtually). The combination of human computer interaction and gamification may have an important impact on the engagement, loyalty and productivity of this group. Due to their familiarity with technology the self-efficacy and social capital of Y generation individuals can be achieved through both virtual and physical rewards (Barreneche, 2012). Rewards can include access to additional informational support and elements of fun (Brox et al., 2011; Bosworth, 2012).

### Amusement factors (fun elements)

Workplace activities can be made more attractive through the introduction of elements of fun (Coller and Scott, 2009; Crookall, 2010; Deterding et al., 2011; Michaelides, 2011; Gomes et al., 2012). A fun environment could be achieved by focusing on a balance between collaboration and competition oriented activities (Fernandes et al., 2012; Gomes et al., 2012). Such programs can result in improved morale, productivity and health behaviors (Bosworth, 2012; Gomes et al., 2012) with the long term outcome of a healthier partnership between employers and employees, a partnership which may result in both individual and organizational benefits.

### Transformative

A gamified system can use a balanced and attractive combination of competition and collaboration in order to transform existing work processes within an organization (Bosworth, 2012; Gomes et al., 2012; Perryer et al., 2012). For example, a gamified work process may allow employees to adopt a new persona, even if only for a limited time. Such a persona can be a different position than their usual one (i.e., Environmental Scientist instead of Project manager) (Fennewald and Kievit-Kylar, 2012). A gamified process could integrate professionalism and moral decisions into the mix in order to enhance the desired skills of the participants (Coller and Scott, 2009). In the short term, the goal of a gamified workplace may be to train employees in new work processes. Medium term goals may be to enhance productivity. The long term goal may be to foster employee and organizational wellbeing.

### Wellbeing-oriented

It has been documented that gamified systems could improve health and productivity while reducing health care related costs (Bosworth, 2012; Ferguson, 2012; Gomes et al., 2012; Lenihan, 2012).

The focus of a gamified system must be on the users' psychosocial experience and wellbeing (Gomes et al., 2012). In regards to psychosocial experiences it is important to recognize that adults have a greater need to document their memories, connections and reactions, thus they can become co-creators of the gamified workplace with the help of modern technology (Barreneche, 2012). This can be particularly powerful for social capital development.

Furthermore, gamification could contribute to the wellbeing of employees and to the wellbeing of management (Kark, 2011). For example, games can be used within an organization for the professional development of managers who could make decisions and identify the consequences of those decisions in a safe environment through simulation and role-play (Kark, 2011).

### Research-generating

Designing, implementing and evaluating a gamified system is a complex task (Brox et al., 2011). For an organization to consider gamifying its work processes, it needs a clear understanding of the potential benefits through research. Gamified systems could transform work related processes at multiple levels including the development of a research oriented culture. Gamified workplaces could allow researchers, managers and employees to collaborate in order to better understand the changes required in their work (Barreneche, 2012). Furthermore, human computer interaction research and practice could contribute to and could benefit a great deal from the gamified workplace in both small and large organizational settings (Gomes et al., 2012).

### Knowledge-based

Finally, gamified processes must be based on knowledge, either as an outcome or as feedback. Successful games have short, medium, and long term goals (Dorling and McCaffery, 2012). The goals of a game are often clear and easy to monitor (Coller and Scott, 2009). In a gamified process some key goals could be setup in a clear manner. Such goals must encourage some level of risk taking, yet the options or leverages available to the employee could be limited to a predetermined set (Farhangi, 2012) such as workplace regulations and policies. Systematic feedback, including rewards, could be used to generate desired behavioral changes.

For example, a gamified process would provide immediate feedback and allow for immediate progression if the employees measurable results meet a given threshold. Furthermore, gamified processes could provide multiple types of feedback such that the employee has incentives to return and practice until they reach the maximum level of achievement possible on each task or combination of tasks (Dorling and McCaffery, 2012), thus placing the employee at the center of the gamified process (I orientation).

## Future directions

The I PLAY AT WORK principles could be used in the future to foster and study productivity in the gamified workplace, health promotion in the gamified workplace, psychological benefits of the gamified workplace and human computer interaction in the gamified workplace. Productivity is one of the key measurements that management is interested in and thus should come first (Kark, 2011). Second, preventative health care can play a major role in reducing the direct and indirect costs of illness in the workforce. Some authors estimate that more than two trillion dollars could be saved in the US alone if prevention is given appropriate attention (Bosworth, 2012). The most promising directions at the moment are gamified systems which include social elements. They seem to work best on widely accepted health behaviors such as weight loss through better nutrition and increased physical activity. However there is limited information on gamified initiatives that focus on less accepted behavior such as alcohol misuse or less understood health conditions such as mental health and illness. It is in this area that psychological research could make a great contribution. Simply put, happy people may be more productive people.

I PLAY AT WORK principles are a work in progress. However, we do hope that they will be useful in generating direction, discussion, and development in relationship to the gamified workplace.

## Conclusion

We are still in the early stages of understanding how gamification can be best used in the workplace for positive behavior change. An analysis of research literature across disciplines identified an emerging base of evidence that suggests gamification as a promising strategy for promoting loyalty, productivity, and wellbeing in the workplace. However there are many gray areas left to address in the near future, such as how to practically and easily measure the value of the gamified workplace for personal and organizational wellbeing. In addition to the value of mapping the present for the benefit of the future, there is also considerable value in reshaping core ideas related to the workplaces. Gamified workplaces can provide opportunities for a more vigorous and strategic inter-disciplinary research agenda that can stimulate investments in the area.

### Conflict of interest statement

The authors declare that the research was conducted in the absence of any commercial or financial relationships that could be construed as a potential conflict of interest.
